# *IDH* mutation status is associated with a distinct hypoxia/angiogenesis transcriptome signature which is non-invasively predictable with rCBV imaging in human glioma

**DOI:** 10.1038/srep16238

**Published:** 2015-11-05

**Authors:** Philipp Kickingereder, Felix Sahm, Alexander Radbruch, Wolfgang Wick, Sabine Heiland, Andreas von Deimling, Martin Bendszus, Benedikt Wiestler

**Affiliations:** 1 Department of Neuroradiology, University of Heidelberg Medical Center, Heidelberg, Germany; 2 Department of Neuropathology, University of Heidelberg Medical Center, Heidelberg, Germany; 3 German Cancer Consortium (DKTK), Clinical Cooperation Unit Neuropathology, German Cancer Research Center (DKFZ), Heidelberg, Germany; 4 Neurology Clinic, University of Heidelberg Medical Center, Heidelberg, Germany; 5 DKTK, Clinical Cooperation Unit Neurooncology, DKFZ, Heidelberg, Germany; 6 DKFZ, Department of Radiology, Heidelberg, Germany

## Abstract

The recent identification of *IDH *mutations in gliomas and several other cancers suggests that this pathway is involved in oncogenesis; however effector functions are complex and yet incompletely understood. To study the regulatory effects of *IDH* on *hypoxia-inducible-factor 1-alpha* (*HIF1A*), a driving force in hypoxia-initiated angiogenesis, we analyzed mRNA expression profiles of 288 glioma patients and show decreased expression of *HIF1A* targets on a single-gene and pathway level, strong inhibition of upstream regulators such as *HIF1A* and downstream biological functions such as angio- and vasculogenesis in *IDH* mutant tumors. Genotype/imaging phenotype correlation analysis with relative cerebral blood volume (rCBV) MRI – a robust and non-invasive estimate of tumor angiogenesis – in 73 treatment-naive patients with low-grade and anaplastic gliomas showed that a one-unit increase in rCBV corresponded to a two-third decrease in the odds for an *IDH* mutation and correctly predicted *IDH* mutation status in 88% of patients. Together, these findings (1) show that *IDH* mutation status is associated with a distinct angiogenesis transcriptome signature which is non-invasively predictable with rCBV imaging and (2) highlight the potential future of radiogenomics (i.e. the correlation between cancer imaging and genomic features) towards a more accurate diagnostic workup of brain tumors.

Somatic mutations of the isocitrate dehydrogenase (*IDH*) 1 and 2 genes are highly frequent in lower-grade (WHO grades II-III) gliomas^1^ and secondary glioblastomas^2^ and were found to be an early event in gliomagenesis^3^ with profound effects on the molecular and genetic route of oncogenic progression^4^,^5^ and on clinical outcome^6^,^7^,^8^. *IDH* mutations were also observed in several other cancers, including acute myeloid leukemia^9^, T-cell lymphoma^10^, chondrosarcoma, enchondroma^11^, and cholangiocarcinoma^12^. The identification of *IDH* mutations in multiple cancers suggests that this pathway is involved in oncogenesis.

Mechanistically, *IDH* mutations induce a neomorphic enzyme activity converting α-ketoglutarate (*α-KG*) to (R)-2-hydroxyglutarate (*(R)-2HG*)^13^. One model for how mutant *IDH* may contribute to oncogenesis and the one that has perhaps gained the most traction is transformation by *α-KG* antagonism^48^. *(R)-2HG* is structurally and chemically very similar to *α-KG* and it has been proposed that *(R)-2HG* competitively inhibits the function of *α-KG-*dependent dioxygenases, such as the JmjC domain-containing histone demethylases (JmjC-KDMs), which cause histone demethylation^5^,^14^,^15^, and the ten-eleven translocation *(TET)* family of DNA hydroxylases, which cause DNA demethylation^5^,^16^ and are considered a major mechanism in the tumorigenesis of *IDH* mutant gliomas^4^,^17^.

Beside these epigenetic modications, *(R)-2HG* has also been shown to regulate the activity of *α-KG*-dependent dioxygenases, specifically *EGLN* prolyl 4-hydroxylases, that are responsible for targeting hypoxia-inducible factor 1-alpha *(HIF1A)* – a driving force in tumorigenesis in general and angiogenesis in particular^18^ – for ubiquitination by the von Hippel-Lindau *(VHL)* tumor suppressor and proteasomal degradation^19^. However, unlike the general assumption that *(R)-2HG* would competitively inhibit the activity of *EGLN* prolyl 4-hydroxylases and thus would lead to increased levels of *HIF*1A, it has on the contrary been shown that *(R)-2HG* acts as an activator rather than an inhibitor of *EGLN* prolyl 4-hydroxylases, ultimately leading to decreased levels of *HIF1A*^20^,^21^,^22^. Although these results were initially surprising, it suggests that low levels of *HIF1A* suppress specific hallmarks of cancer required for aggressive behaviour (such as angiogenesis) in *IDH* mutant gliomas^20^,^23^ which fits with the indolent clinical course of these tumors^6^,^7^,^8^.

The aim of the present study was to study the regulatory effects of *IDH-1/2* on *HIF1A* and related downstream signaling with mRNA expression and functional analysis and subsequent genotype / imaging phenotype correlation analysis to assess whether the observed molecular findings translate into distinct phenotypes, which can be detected non-invasively on MRI and thus would allow prediction of the *IDH* mutation status.

## Results

**Figure 1:**
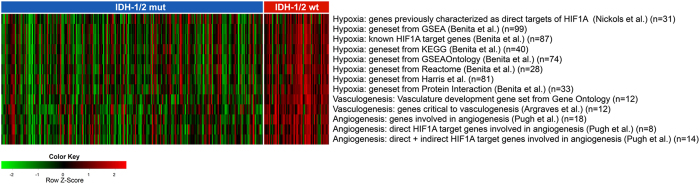
Gene set variation analysis (GSVA) of differentially activated gene sets identified inhibition of hypoxia, vasculo- and angiogenesis signaling pathways in *IDH*-1/2 mutant tumors and activation in *IDH*-1/2 wild-type tumors. Rows represent samples of 288 patients with low-grade diffuse or anaplastic gliomas from The Cancer Genome Atlas (TCGA). Columns represent GSVA enrichment scores for the individual gene set.

IPA of upstream regulators revealed significant inhibition of *HIF1A* in *IDH-1/2* mutant tumors (Table 1a and Supplementary Table 3) and correspondingly increased expression of *EGLN* prolyl 4-hydroxylases – specifically *EGLN 1* and *EGLN3* which are the principal prolyl hydroxylases for degradation of *HIF1A*^24^ – on a single-gene level (Supplementary Fig. 1). Moreover both IPA of upstream regulators and single-gene analysis (Table 1a and Supplementary Fig. 1) concordantly demonstrated significantly decreased expression of *HIF1A*-regulated pro-angiogenic key targets such as vascular endothelial growth factor A *(VEGFA)*, angiopoietin-2 (*ANGPT2*) and platelet-derived growth factor A *(PDGFA).* We then studied the differential activity of *HIF1A* dependent pathways between *IDH-1/2* mutant and wild-type tumors using a novel method for assessing the enrichment of gene sets in individual samples called gene set variation analysis (GSVA)^25^. This approach evaluates the enrichment of specific gene sets within the individual samples rather than on a group-level, which is a distinct advantage over gene set enrichment analysis. GSVA unveiled highly differential expression of *HIF1A* dependent gene sets, with inhibition of hypoxia-mediated signaling in general, and specifically vasculo- and angiogenesis in *IDH-1/2* mutant tumors (Fig. 1; empirical Bayes moderated-t p-values for individual gene sets are provided in Supplementary Table 2). These findings were further supported by the IPA downstream effects analysis, which demonstrated a significant inhibition of *HIF1A* mediated pro-angiogenic biological functions such as development, migration and movement of endothelial cells, development of blood vessel, vasculogenesis, angiogenesis, and (neo)vascularization in *IDH-1/2* mutant tumors (Table 1b and Supplementary Table 3).

**Figure 2:**
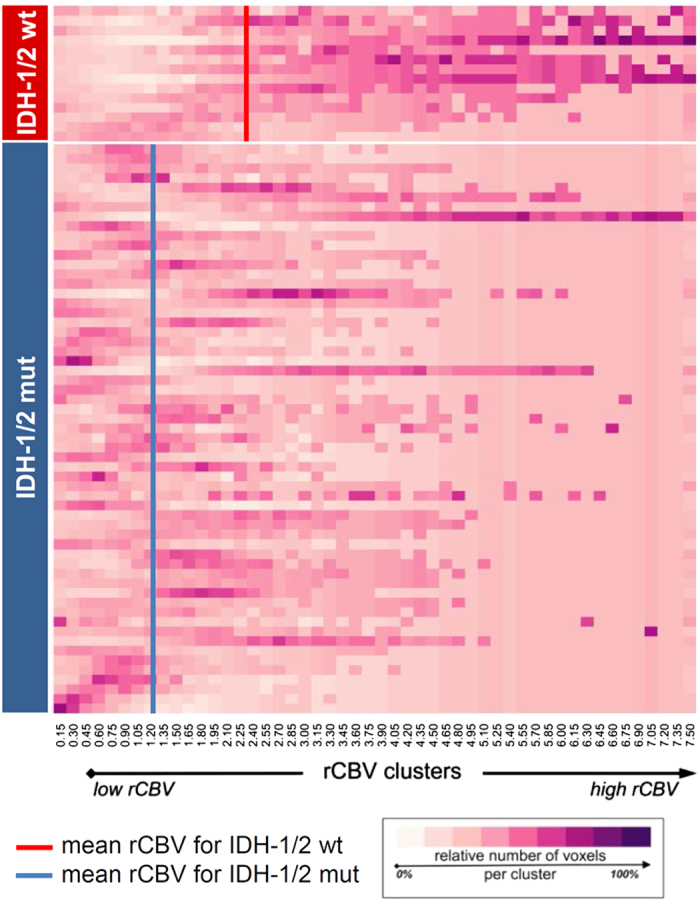
Relative cerebral blood volume (rCBV) imaging heat-map of 73 treatment-naive patients with low-grade diffuse and anaplastic gliomas. The color of the voxel corresponds to the relative number of rCBV-voxels in each cluster and goes from white (low frequency) to purple (high frequency). Overall, *IDH-1/2* wild-type tumors (top of the heat-map) clustered at a significantly higher rCBV as compared to *IDH-1/2* mutant tumors (bottom of the heat-map) (see Table 2 for statistical results).

**Figure 3:**
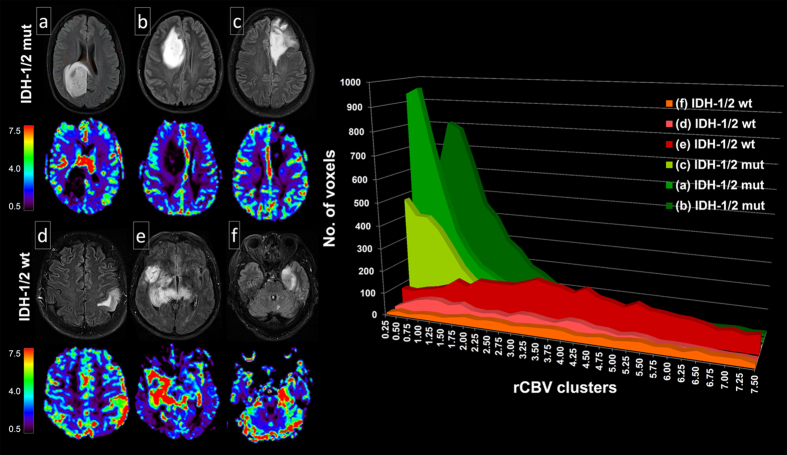
Pre-treatment MRI (FLAIR and corresponding rCBV images) of six representative patients with *IDH-1/2* mutant (**a**–**c**) and wild-type (**d**–**f**) gliomas. Histogram analysis demonstrates the distribution of rCBV-voxels, with *IDH-1/2* mutant tumors clustering at substantially lower values as compared to their wild-type counterpart.

To assess whether these differential genetic signatures between *IDH-1/2* mutant and wild-type tumors translate into distinct phenotypes, which can be detected non-invasively, rCBV imaging – a robust and clinically meaningful estimate of tumor angiogenesis^26^,^27^,^28^ – was analyzed from a local dataset in 73 treatment-naive patients with low-grade diffuse and anaplastic gliomas. Using a semi-automated radiogenomic imaging approach (see Methods) we demonstrated that *IDH-1/2* mutant and wild-type tumors are both associated with distinct imaging phenotypes, with *IDH-1/2* mutant tumors clustering at a significantly lower rCBV (median of the 50^st^ percentile, 1.09; interquartile range, 0.80–1.47) as compared to their wild-type counterpart (median of the 50^st^ percentile, 2.08; interquartile range, 1.49–2.57), suggesting indeed decreased angiogenesis in *IDH-1/2* mutant tumors as was predicted from the IPA downstream effects analysis of gene expression data (Fig. 2; representative cases are shown in Fig. 3). In detail, rCBV was significantly different between *IDH-1/2* mutant and wild-type tumors for all evaluated percentiles, with the 90^st^ percentile yielding the highest z-score (4.89; p < 0.01) (Table 2). To quantify the predictive value of rCBV for determining *IDH-1/2* mutation status we analyzed the cumulative rCBV histogram distribution with cross-validated logistic-regression models. Thereby, the highest AUC was again obtained for the 90^st^ percentile of the cumulative rCBV histogram distribution (92% (95% CI, 84–100%)) where a one-unit increase in rCBV corresponded to a decrease in the odds for presence of an *IDH-1/2* mutation by 69% and correctly predicted *IDH-1/2* mutation status in 88% of patients (positive predictive value (PPV): 89%, negative predictive value (NPV): 78% – detailed results are available in the Table 2).

## Discussion

The recent discovery that genes encoding *IDH-1/2* are recurrently mutated in several cancers, which induce a neomorphic enzyme activity that results in the production of the oncometabolite *(R)-2HG*, unveiled a fascinating and complex biology behind the dysregulation and functional consequences of metabolites in cancers^29^. However, the resulting biological effects of *IDH* mutation and *(R)-2HG* accumulation remain controversial, exemplified in the case of *HIF1A* signaling, a major oncogenic pathway in malignant glioma. While some studies demonstrated that *IDH* mutation increases *HIF1A* levels 30,31, several others reported opposite results^20^,^21^,^22^: Koivunen *et al.* found *EGLN* activation in response to *(R)-2HG* accumulation, in turn leading to increased *HIF1A* degradation^20^. More recently, many *HIF1A* target genes were shown to be lower expressed in *IDH* mutant gliomas^32^. In line with the latter reports, the results from the present study show that *IDH* mutant tumors are characterized by an increased expression of *EGLN* prolyl 4-hydroxylases that mark *HIF1A* for polyubiquitylation and proteasomal degradation, leading to decreased *HIF1A* activation and downstream inhibition of hypoxia, vasculo- and angiogenesis related signaling. These signaling pathways are a prerequisite for aggressive tumor behavior which is underscored by the relative indolent course of low-grade and anaplastic gliomas carrying *IDH* mutations, as compared to their wild-type counterpart which usually carry a dismal prognosis similar to those of glioblastoma^33^. Most importantly, our semi-automatic radiogenomic imaging approach showed that these differential molecular signatures between *IDH* mutant and wild-type tumors translate into distinct phenotypes which are non-invasively predictable with rCBV imaging in a clinical setting. The prognostic value of rCBV imaging has already been shown before by Law *et al.*^34^, who reported that lower rCBV translates into significantly prolonged time to progression in 189 glioma patients. While these authors did not account for *IDH*, it seems reasonable to assume that this finding indeed reflects *IDH* status, given that in their study the proportion of tumors with high rCBV considerably increases with tumor grade, as does the percentage of *IDH* wild type tumors in general^35^.

Despite the straightforward results of the present study, we however have to acknowledge several limitations. First, differential mRNA expression and genotype/imaging phenotype analyses were performed in two independent cohorts (The Cancer Genome Atlas (TCGA) and local dataset), thus we were not able to directly define an mRNA expression correlate for the analyzed rCBV samples, and instead used the *IDH* mutation status as a connector between the TCGA and local dataset. Second, we were not able to determine whether decreased angiogenesis in *IDH* mutant tumors is solely a consequence of increased expression of *EGLN* prolyl 4-hydroxylases and subsequent decreased *HIF1A* activation or whether epigenetic modifications in *IDH* mutant tumors that result in reorganization of the methylome and gene expression may have also had implications on angiogenesis.

**Figure 4:**
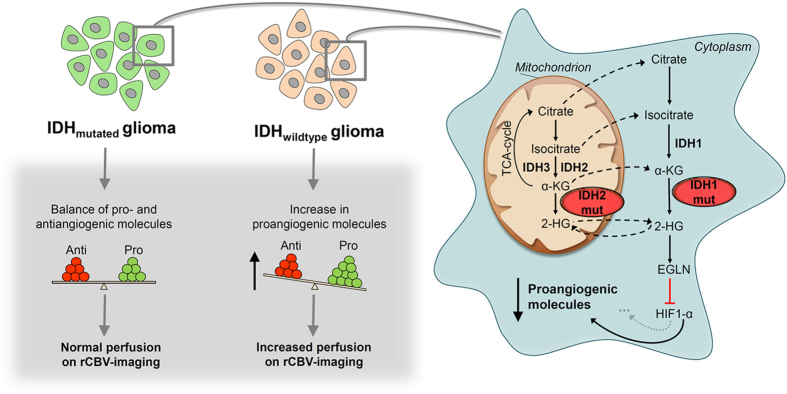
Synopsis of how *IDH* mutation status is linked to angiogenesis and alteration in rCBV on DSC-MRI in human glioma based on this hypothesis generating study. In brief, mutations in cancer-associated *IDH* acquire neoactivity producing 2-hydroxyglutarate *(2HG)*. *(R)-2HG* potentiates *EglN* activity that mark the hypoxia inducible factor *HIF1A* for polyubiquitylation and proteasomal degradation, leading to decreased *HIF1A* activation in *IDH* mutant tumors, compared with their wild-type counterparts. This results in a distinct transcriptome signature induced by upregulation of hypoxia, vasculo- and angiogenesis related signaling pathways in *IDH* wild-type tumors, which is non-invasively detectable with rCBV imaging.

In conclusion (Fig. 4), these findings (1) show that *IDH* mutation status is associated with a distinct angiogenesis transcriptome signature which is non-invasively predictable with rCBV imaging and (2) highlight the potential future of radiogenomics (i.e. the correlation between cancer imaging and genomic features) towards a more accurate diagnostic workup of brain tumors.

## Methods

### Analysis of differential mRNA expression (TCGA dataset)

RNA sequencing raw data (mapped to genes) and curated *IDH-1/2* mutation data were downloaded from TCGA data portal on October 15^th^, 2014. In total, RNA sequencing and curated mutation data was available for 288 unique samples with low-grade diffuse (WHO °II) or anaplastic (WHO °III) gliomas (detailed sample information is provided in Supplementary Table 1) which were then analyzed using Bioconductor 3.0^36^, a package implemented in R 3.1.2 (R Foundation for Statistical Computing, Vienna, Austria)^37^. First, normalization and differential gene expression analysis of RNA sequencing counts was performed using the edgeR package^38^, which assumes a negative binomial distribution of count data, filtering lowly expressed transcripts.

To study relative pathway activity on the level of individual samples, we used the GSVA package^25^. Using a nonparametric approach, GSVA transforms a gene-by-sample gene expression matrix into a gene set–by-sample gene set enrichment matrix, facilitating the identification of differentially activated genesets for each sample. GSVA was performed on 17 gene sets related to hypoxia, vasculo- and angiogenesis (a detailed listing is provided in Supplementary Table 2) identified through the Molecular Signatures Database (MSigDB - www.broadinstitute.org/gsea/msigdb)^39^ and literature research. Next, empirical Bayes moderated t statistics^40^ were calculated, and gene sets with FDR-q < 0.05 were considered to have significantly differential activity between samples with mutant and wild-type *IDH-1/2* tumors. Of these gene sets, individual GSVA enrichment scores were plotted as a heatmap with the gplots package.

Furthermore, the differential upstream biological causes and probable downstream effects between mutant and wild-type *IDH-1/2* tumors were analyzed with the proprietary Ingenuity Pathway Analysis (IPA, QIAGEN, Redwood City, CA, USA) using a fold change filter of |1.5| and FDR-q < 0.05. Briefly, the software calculates both an overlap p value (based on Fisher’s exact test) and an activation z score, which is based on the expression state of activating and inhibiting genes, for manually curated upstream regulators and downstream biological functions. Only results with both an FDR-q < 0.05 and a z score > |2| were considered significant.

### Analysis of MRI data (local dataset)

The local ethics committee of the University of Heidelberg approved retrospective data evaluation, and the requirement for patient informed consent was waived (ethics approval number: S-320/2012). Data evaluation was performed in accordance with relevant guidelines and regulations. Pre-treatment MRI scans of all adult patients (age ≥18 years) with newly diagnosed, pathologically confirmed low-grade diffuse (WHO grade II) or anaplastic (WHO grade III) gliomas in the period of July 2009 and April 2015 were screened. Out of these, dynamic susceptibility contrast-enhanced (DSC) and conventional MRI was available for 73 individual patients (detailed sample information is provided in Supplementary Table 1), which were then selected for subsequent analysis.

Images were acquired in the routine clinical workup using a 3 Tesla MR system (Magnetom Verio/Trio TIM, Siemens Healthcare, Erlangen, Germany) with a 12-channel head-matrix coil. Acquisition of the DSC-MRI sequence was performed as described previously^41^. In brief, prior dynamic imaging, a 0.1 mmol/kg prebolus dose of gadoterate meglumine (Gd-DOTA, DOTAREM, Guerbet, France) was administered to diminish T1 effects that might result from agent extravasation. DSC-PWI was obtained with a T2*-weighted gradient-echo EPI sequence during the bolus injection of a standard dose (0.1 mmol/kg) of intravenous gadoterate meglumine. Twenty-six to 28 slices with a thickness of 5 mm were acquired with spectral fat suppression (TE = 36 ms, TR = 2220 ms, FA = 90°, field of view = 240 mm × 240 mm, image matrix = 128 × 128). In total, 50 to 75 dynamic measurements were performed. Fluid attenuated inversion recovery (FLAIR) images were acquired with the following parameters: TI = 2400 ms; TE = 85 ms; TR = 8500 ms; section thickness, 5 mm; interslice gap, 5%.

Post-processing of DSC-MRI and FLAIR data was performed with dedicated software (Olea Sphere v 2.3, Olea Medical, La Ciotat, France). First, a rigid-body registration was applied to the DSC-MRI data to correct for motion artifacts. An arterial-input function (AIF) was determined automatically using cluster analysis techniques^42^ and deconvolution of the AIF was performed using a time-insensitive block-circulant singular value decomposition (cSVD)^43^. Whole-brain relative CBV (rCBV) maps were generated by using an established tracer kinetic model applied to the first-pass data, with rCBV values computed pixel-by-pixel as the area under the concentration time curve (AUC) divided by the AUC of the AIF^44^. Mathematical correction of the contrast agent leakage from the intravascular to extracellular space was performed with the method suggested by Boxerman *et al.*^45^. Next, the FLAIR-hyperintense tumor was outlined according to a semi-automated region-growing segmentation method, which examines neighboring voxels of an initial seed point/voxel and determines whether the voxel neighbors should be added to the ROI. Automatic iteration of this process was performed on each slice of the FLAIR-images until the hyperintense FLAIR abnormality (excluding those resulting from obvious leukariosis) was included in the ROI. Automatic co-registration of rCBV maps with corresponding FLAIR images which included the previously segmented FLAIR ROIs was performed prior voxel-wise extraction of rCBV values from the segmented tumor. The extracted rCBV voxel values from individual patients were then distributed into 50 equally spaced clusters (width of 0.15) for rCBV-values in the range of 0.0 to 7.5. Finally, the relative number of rCBV-voxels in each cluster were plotted in R as a heatmap with the gplots package. Separate extraction and analysis of rCBV voxels from the contrast-enhancing tumor parts was not performed since this was only present in a small minority of patients included in the present study.

The rCBV histogram distribution was further analyzed to evaluate whether rCBV is different between patients *IDH-1/2* mutant and wild-type tumors. We evaluated the significance of several percentiles (5^th^, 10^th^, 25^th^, 50^st^, 75^th^, 90^st^, 95^th^, 99^th^) from the rCBV histogram distribution with a Mann-Whitney U test. Furthermore, leave-one-out cross-validated logistic regression analysis was performed to assess the significance of each rCBV percentile for predicting *IDH-1/2* mutation status (using a cut-off of the predicted probability of 0.5). P-values < 0.05 were considered significant.

### *IDH* mutation status assessment (local dataset)

*IDH-1/2* mutation screening was performed for all evaluated patients (n=73) with immunohistochemistry (IHC) for *IDH1-R132H* and DNA sequencing for IHC-negative cases. Tissue was provided by the Department of Neuropathology, Institute of Pathology, University of Heidelberg Medical Center, Germany in accordance with local ethical approval. All experiments were performed in accordance with relevant guidelines and regulations.

IHC of formalin-fixed paraffin-embedded tumor tissue for *IDH1-R132H* was performed as described previously^46^. In brief, sections cut to 3 μm were incubated and processed on a Ventana BenchMark XT® immunostainer (Ventana Medical Systems, Tucson, AZ, USA). Antibodies were anti-human *IDH1-R132H* (H09, Dianova, Hamburg, Germany) The Ventana staining procedure included pretreatment with cell conditioner 2 (pH 6) for 60 min, followed by incubation with primary antibody at 37 °C for 32 min. Incubation was followed by Ventana standard signal amplification, UltraWash, counterstaining with one drop of hematoxylin for 4 min and one drop of bluing reagent for 4 min. For visualization, ultraView™ Universal DAB Detection Kit (Ventana Medical Systems) was used. *IDH1-R132H* staining was evaluated as described previously^47^.

DNA sequencing for IHC-negative cases was performed as described previously^1^. In brief, DNA-extraction from paraffin-embedded tumor was performed tissue using the DNeasy blood and tissue kit (Qiagen, Hilden, Germany). A fragment of 129-bp length spanning the catalytic domain of *IDH1* including codon 132 was amplified using sense primer *IDH*1f CGGTCTTCAGAGAAGCCATT and antisense primer *IDH*1r GCAAAATCACATTATTGCCAAC, and a fragment of 150 bp length spanning the sequence encoding the catalytic domain of *IDH2* including codon 172 was amplified using the sense primer *IDH*2f AGCCCATCATCTGCAAAAAC and antisense primer *IDH*2r CTAGGCGAGGAGCTCCAGT. Sequences were determined using a semiautomated sequencer (ABI 3100 Genetic Analyzer, Applied Biosystems, Foster City, CA) and the Sequence Pilot version 3.1 (JSI-Medisys, Kippenheim, Germany).

## Additional Information

**How to cite this article**: Kickingereder, P. *et al.* *IDH* mutation status is associated with a distinct hypoxia/angiogenesis transcriptome signature which is non-invasively predictable with rCBV imaging in human glioma. *Sci. Rep.* **5**, 16238; doi: 10.1038/srep16238 (2015).

## Supplementary Material

Supplementary Information

Supplementary Table 1

Supplementary Table 2

Supplementary Table 3

## Figures and Tables

**Table 1:** Ingenuity pathway analysis of upstream regulators (A) and downstream biological functions (B).

(A) Upstream regulators in *IDH-1/2* mutant tumors				
Upstream regulator	Molecule type	p value of overlap	Activation z score	Predicted activation state
Vegf	Group	4.11E-30	−7.287	Inhibited
PDGF BB	Complex	3.94E-36	−5.17	Inhibited
*HIF1A*	Transcription regulator	6.54E-10	−4.65	Inhibited
*VEGFA*	Growth factor	1.27E-10	−4.306	Inhibited
Pdgf	Complex	2.94E-10	−3.646	Inhibited
*ANGPT2*	Growth factor	6.79E-09	−3.361	Inhibited
**(B) Downstream biological functions in** ***IDH-1/2*** **mutant tumors**				
**Biological function**		**p value of overlap**	**Activation z score**	**Predicted activation state**
Development of blood vessel		7.87E-30	−4.185	Decreased
Migration of endothelial cells		3.26E-15	−3.799	Decreased
Vasculogenesis		8.89E-28	−3.717	Decreased
Movement of endothelial cells		2.66E-16	−3.540	Decreased
Angiogenesis		9.39E-28	−3.556	Decreased
Neovascularization		1.53E-10	−2.451	Decreased
Vascularization		6.85E-12	−2.319	Decreased
Development of endothelial cells		6.58E-12	−2.020	Decreased

Both concordantly demonstrate a significant decrease in key angiogenic regulators such as *HIF1A, VEGFA, PDGF* or *ANGPT2* (A) and consequently angiogenic biological processes (B) in *IDH-1/2* mutant tumors. Full results are available in the data supplement (Supplementary Table 3).

**Table 2:** rCBV histogram analysis for differentiating *IDH* *-1/2* mutant and wildtype tumors.

Percentile	Mann-Whitney U test		Logistic regression							
	p-value	z-score	OR	(95% CI)	p-value	AUC	(95% CI)	Correctly classified	PPV	NPV
5	0.00	3.46	0.07	(0.01–0.94)	0.05	66.7%	(50.2%–83.2%)	82.2%	82.9%	66.7%
10	0.05	1.93	0.08	(0.01–0.55)	0.01	71.6%	(55.8%–87.3%)	83.6%	84.1%	75.0%
15	0.01	2.49	0.08	(0.01–0.43)	0.00	74.8%	(60.1%–89.6%)	82.2%	83.8%	60.0%
25	0.00	2.87	0.09	(0.02–0.41)	0.00	79.9%	(67.5%–92.3%)	82.2%	83.8%	60.0%
50	0.00	4.11	0.17	(0.04–0.76)	0.02	85.5%	(75.2%–95.8%)	83.6%	86.2%	62.5%
75	0.00	4.40	0.23	(0.06–0.88)	0.03	88.0%	(77.2%–98.9%)	86.3%	87.7%	75.0%
**90**	**0.00**	**4.89**	**0.31**	**(0.13**–**0.78)**	**0.01**	**92.2%**	**(84.3%**–**100.0%)**	**87.7%**	**89.1%**	**77.8%**
95	0.00	4.88	0.40	(0.22–0.75)	0.00	92.2%	(84.8%–99.7%)	86.3%	88.9%	70.0%
99	0.00	4.72	0.56	(0.42–0.76)	0.00	90.8%	(83.9%–97.7%)	82.2%	85.9%	55.6%

Abbreviations: AUC = Area under the curve; CI = confidence interval; NPV = negative predictive value; OR = Odds ratio; PPV = positive predictive value.
