# Supplementary material for: IDH mutation status is associated with a distinct hypoxia/angiogenesis transcriptome signature which is non-invasively predictable with rCBV imaging in human glioma
Source: Sci Rep. 2015 Nov 5;5:16238. doi: 10.1038/srep16238 (PMC4633672; doi:10.1038/srep16238)

Supplementary Data

IDH-mutation status is associated with a distinct hypoxia/angiogenesis transcriptome signature which is non-invasively predictable with rCBV-imaging in human glioma.

Philipp Kickingereder MD[1], Felix Sahm MD[2,3], Alexander Radbruch MD[1,6], Wolfgang Wick MD[4,5], Sabine Heiland PhD[1], Andreas von Deimling MD[2,3], Martin Bendszus MD[1], and Benedikt Wiestler MD[1,4,5]

(1) Department of Neuroradiology, University of Heidelberg Medical Center, Heidelberg, Germany
(2) Department of Neuropathology, University of Heidelberg Medical Center, Heidelberg, Germany
(3) German Cancer Consortium (DKTK), Clinical Cooperation Unit Neuropathology, German Cancer Research Center (DKFZ), Heidelberg, Germany
(4) Neurology Clinic, University of Heidelberg Medical Center, Heidelberg, Germany
(5) DKTK, Clinical Cooperation Unit Neurooncology, DKFZ, Heidelberg, Germany
(6) DKFZ, Department of Radiology, Heidelberg, Germany

Supplementary Figure 1. Gene expression heat-map of key *HIF1A* target genes showing increased expression of key angiogenic regulators such as *VEGFA*, *ANGPT* or *PDGF* in *IDH-1/2* wild-type tumors and on the contrary, increased expression of *EglN 1* and *3* – responsible for degradation of *HIF1A* – in *IDH-1/2* mutant tumors. Columns represent samples of 288 patients with low-grade diffuse or anaplastic gliomas from The Cancer Genome Atlas (TCGA). The statistical track at the bottom shows the logarithmic plot of p-values for each genomic position, with colored bars indicating either significantly increased expression of the genomic location for *IDH-1/2* wild-type (red) or mutant (blue) tumors.

Supplementary Table 1. Detailed information on histology and *IDH* mutation status from both TCGA and local dataset. (Excel file)

Supplementary Table 2. Gene sets evaluated for differential mRNA-expression between *IDH-1/2* mutated and wild-type tumors in the gene set variation analysis (GSVA). (Excel file)

Supplementary Table 3. Ingenuity pathway analysis of upstream regulators and downstream biological functions. (Excel file)

Supplementary Figure 1.

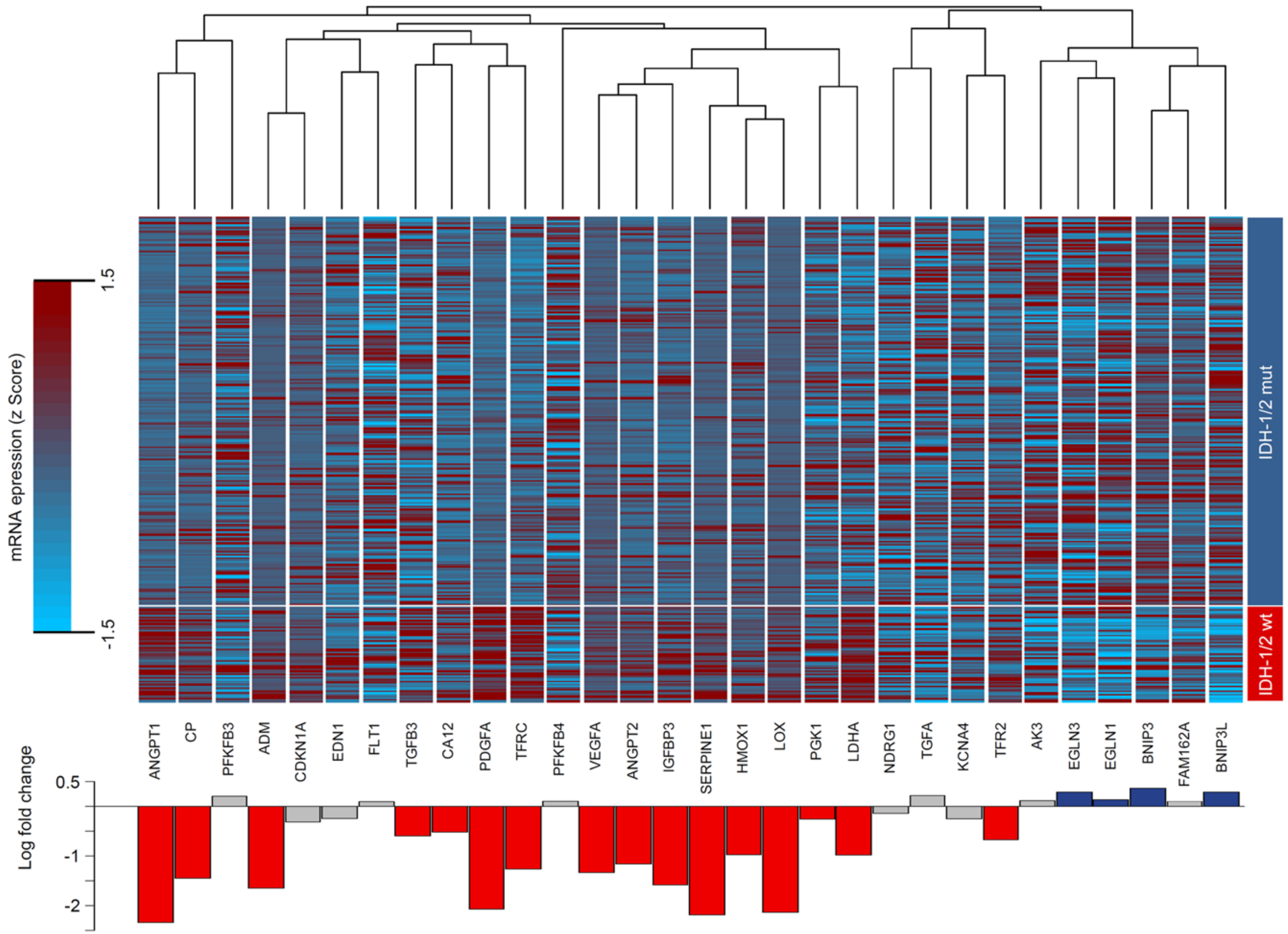

Supplement: Supplementary Information [file srep16238-s1.pdf]
